# Drug-based vector control: a potential new paradigm

**DOI:** 10.1186/s12936-017-1835-7

**Published:** 2017-05-17

**Authors:** Pedro Alonso, Dirk Engels

**Affiliations:** 1 Global Malaria Programme, World Health Organization, Geneva, Switzerland; 2 Control of Neglected Tropical Diseases, World Health Organization, Geneva, Switzerland

With the vision of a malaria free world, the Global Technical Strategy for malaria 2016–2030 [1] sets ambitious, but attainable goals for the next 14 years. Based on lessons from the 1953 Global Malaria Eradication Programme, where leadership declared that all of the tools needed were at hand [2], the GTS instead highlights that innovation will need to play an important role in achieving key milestones towards malaria eradication [3]. This includes the development of new and improved tools, optimizing the use of currently available ones and enabling the rapid uptake of emerging tools, interventions and strategies [1].

Vector control, historically a key pillar of the hard-won achievements against malaria, is now challenged by insecticide resistance [4, 5]. The impact on public health is difficult to document, and there is recent evidence from a multi-centre trial that long-lasting insecticide-treated nets remain effective in spite of variable levels of resistance to pyrethroïd insecticides [6]. However it is clear that malaria vector control, as well as interventions based on medicines, are inherently driving towards resistance, and thus both risk mitigation strategies and replacement technologies must be planned for. Furthermore, success in vector control has led to the recognition that residual transmission, mostly by outdoor biting mosquitoes, represents a significant challenge to malaria elimination efforts [7].

A new drug-based vector control strategy with ivermectin, has shown the potential to become a supplementary intervention, acting as an endectocide, a medicine causing the death of Anopheles mosquitoes when ingested in sufficient doses in a blood meal [8]. Ivermectin is an antiparasitic used extensively in mass drug administration for the control of onchocerciasis and lymphatic filariasis (LF) There is an emerging body of work supporting this potential novel use of ivermectin, to potentially impact malaria transmission if used in mass drug administration.

If evidence is obtained that mass administration with ivermectin can have a safe, incremental and cost-effective impact on malaria transmission, then this new intervention could play a role in the reduction of malaria and neglected tropical diseases given the major overlap of these diseases in the tropics.

In 2016, the Global Malaria Programme (GMP) and the Department for Control of Neglected Tropical Diseases (NTD) jointly constituted a WHO Technical Consultation to evaluate the emerging evidence on this application of ivermectin and to develop a target product profile of an endectocide as malaria vector control tool. This will provide guidance to the scientific community on the kind of evidence required to develop a WHO policy recommendation on the use of ivermectin or other endectocides for malaria transmission control. The main conclusions of the consultation [9] were presented to the Malaria Policy Advisory Committee (MPAC) in September 2016. MPAC provided input on the TPP and the required evidence needed to consider a policy recommendation (clearly not on the potential intervention itself) [10]. The final TPP has been revised and is available on WHO website. We believe that this exercise of identifying the main data gaps and priorities needed for a policy recommendation on this potential novel tool will help optimize early efforts and investments. Time and a focused research and development effort will tell us whether the repurposing of this old drug, or a new endectocide, may become a new intervention to be used in improved vector control, or even become an elimination tool. At the same time it provides an opportunity also to revise and optimize the large-scale use of ivermectin for NTDs and develop products and strategies that would meet expectations for both programmes.

This Thematic Series in the Malaria Journal is based on the extensive background review performed by the authors and contributed to the preparations and evidence presented at the WHO technical consultation.
